# Differences between *Cryptococcus neoformans* and *Cryptococcus gattii* in the Molecular Mechanisms Governing Utilization of D-Amino Acids as the Sole Nitrogen Source

**DOI:** 10.1371/journal.pone.0131865

**Published:** 2015-07-01

**Authors:** Yun C. Chang, Ami Khanal Lamichhane, James Bradley, Laura Rodgers, Popchai Ngamskulrungroj, Kyung J. Kwon-Chung

**Affiliations:** 1 Molecular Microbiology Section, Laboratory of Clinical Infectious Diseases, NIAID, NIH, Bethesda, MD, United States of America; 2 Department of Microbiology, Faculty of Medicine Siriraj Hospital, Mahidol University, Bangkok, Thailand; University of Minnesota, UNITED STATES

## Abstract

The ability to grow on media containing certain D-amino acids as a sole nitrogen source is widely utilized to differentiate *Cryptococcus gattii* from *C*. *neoformans*. We used the *C*. *neoformans* H99 and *C*. *gattii* R265 strains to dissect the mechanisms of D-amino acids utilization. We identified three putative D-amino acid oxidase (*DAO*) genes in both strains and showed that each *DAO* gene plays different roles in D-amino acid utilization in each strain. Deletion of *DAO2* retarded growth of R265 on eleven D-amino acids suggesting its prominent role on D-amino acid assimilation in R265. All three R265 *DAO* genes contributed to growth on D-Asn and D-Asp. *DAO3* was required for growth and detoxification of D-Glu by both R265 and H99. Although growth of H99 on most D-amino acids was poor, deletion of *DAO1* or *DAO3* further exacerbated it on four D-amino acids. Overexpression of *DAO2* or *DAO3* enabled H99 to grow robustly on several D-amino acids suggesting that expression levels of the native *DAO* genes in H99 were insufficient for growth on D-amino acids. Replacing the H99 *DAO2* gene with a single copy of the R265 *DAO2* gene also enabled its utilization of several D-amino acids. Results of gene and promoter swaps of the *DAO2* genes suggested that enzymatic activity of Dao2 in H99 might be lower compared to the R265 strain. A reduction in virulence was only observed when all *DAO* genes were deleted in R265 but not in H99 indicating a pathobiologically exclusive role of the *DAO* genes in R265. These results suggest that *C*. *neoformans* and *C*. *gattii* divergently evolved in D-amino acid utilization influenced by their major ecological niches.

## Introduction

*Cryptococcus neoformans* and *Cryptococcus gattii* are two closely related basidiomycetous yeasts that cause cryptococcosis in humans and animals [1]. Although these two species share 85–90% genomic identity [2] and cause disease that is treated by the same therapeutic measures, these two species are distinguishable in many ways. The major ecological niche of *C*. *neoformans* is pigeon droppings and distributed world-wide. *C*. *neoformans* causes meningoencephalitis mainly in HIV infected and other immunocompromised patients [1]. The major ecological niche of *C*. *gattii* is higher plants. *C*. *gattii* causes diseases more frequently in immunocompetent patients than in immunocompromised individuals and until the 1990s was known to be prevalent only in subtropical to tropical regions [3,4]. However, the outbreak of *C*. *gattii* infection reported on Vancouver Island, Canada in early 2000 [5,6] suggested that the species had spread to temperate zones and it is now recognized as an emerging pathogen in the Pacific Northwest [7,8].

The yeast cell morphology of these two species is similar although strains of *C*. *gattii* tend to produce more ovoidal to pyriform cells compared to those produced by *C*. *neoformans* which are globose with rare pyriform cells [9]. These two species are clearly discernable in the morphology of their sexual spores. However, production of sexual spores is impractical for routine identification. Prior to the availability of molecular tests, laboratories had used serotyping or biochemical tests to differentiate between the two species. The most widely used biochemical tests relied on differences in nutritional growth properties. For instance, creatinine is used as the sole source of nitrogen by both species but the enzyme, creatinine deiminase, responsible for creatinine utilization, is regulated differently between the two species [10]. Creatinine deiminase is repressed by ammonia in *C*. *neoformans* but not in *C*. *gattii*. This difference is manifested by a change in the color of the diagnostic media as the pH changes [10,11]. *C*. *gattii* utilizes glycine as both carbon and nitrogen sources and a majority of *C*. *gattii* isolates are resistant to L-canavanine while the majority of *C*. *neoformans* strains can utilize glycine only as a nitrogen source but not as a carbon source and are susceptible to L-canavanine [12–14]. As a result, media containing L-canavanine and glycine with bromothymol blue as the color indicator for growth (CGB medium) can efficiently differentiate between these two species [14–16]. Furthermore, most isolates of *C*. *gattii* can use D-proline or D-alanine as the sole nitrogen source and can assimilate D-tryptophan to produce pigment whereas only a minor population of *C*. *neoformans* can do the same [15,17,18]. The mechanisms responsible for these diagnostic tests, however, have not been investigated. It is known that plants can synthesize D-amino acid derivatives and some D-amino acids such as D-alanine are widely reported in higher plants [19,20]. Furthermore, D-amino acids have been found in the mammalian forebrain and various tissues [21–23]. Because the major ecological niche of *C*. *gattii* is plant, the patterns of D-amino acid utilization may bear pathobiological and ecological significance. We, therefore, considered it important to discern the mechanisms underlying the differences between the two species.

We screened for mutants of *C*. *gattii* that grew poorly on D-Ala and D-Pro as the sole nitrogen source. One of the isolated mutants contained an insertion at a gene containing a D-amino acid oxidase domain. D-amino acid oxidase (Dao; EC 1.4.3.3) is a flavin adenine dinucleotide (FAD)-containing enzyme that catalyzes the oxidative deamination of D-amino acids to the corresponding α-keto acids and ammonia (http://www.chem.qmul.ac.uk/iubmb/). Dao has high stereoselectivity towards the D-isomers of amino acids and is almost inactive towards the corresponding L-isomers [24]. Dao plays distinct physiological roles in different organisms [25]. For instance, Dao can catabolize D-amino acids and allow bacteria or yeast to grow using D-amino acids as the sole carbon or nitrogen source. Dao also has a regulatory role in the human brain where it controls the levels of the neuromodulator D-serine.

In this study, we found that the two representative genome-sequenced strains, *C*. *neoformans* (H99) and *C*. *gattii* (R265), each contain three paralogous *DAO* genes. We characterized the different role of each *DAO* gene in D-amino acid utilization. Interestingly, *DAO* genes played a role in pathobiology of *C*. *gattii* but not in *C*. *neoformans*.

## Materials and Methods

### Strains and culture conditions

*C*. *neoformans* H99 and *C*. *gattii* R265 are genome sequenced strains. All other strains derived from *C*. *neoformans* H99 and *C*. *gattii* R265 are listed S1 Table. Strain Y-1091 of *Rhodosporidium toruloides* was obtained from USDA Agricultural Research Service. Spot assays were performed as described [26] on YNB media with 2% glucose and 10 mM ammonium sulfate or 10 mM D- or L-amino acids as the sole nitrogen source. For toxicity tests, 100 mM of the indicated D-amino acid was added to YNB medium with 2% glucose and 10mM ammonium sulfate. Plates were incubated at 30°C for 3–14 days and photographed.

### Screening and identification of the genes important for growth of R265 on D-amino acid medium

A T-DNA insertion library of R265 was made using *Agrobacterium tumefaciens* mediated transformation as described previously [27]. To screen for mutants that grow poorly on D-Ala or D-Pro as the sole nitrogen source, about 20,000 individual transformants were replica spotted on yeast nitrogen base media supplemented with 50 μg/ml geneticin, 200 μM cefotaxime and containing either 10 mM ammonium sulfate, D or L-Ala, D or L-Pro as the sole nitrogen source. The plates were incubated at 30°C and growth was monitored for 7 days. Mutants that grew poorly on D-Ala or D-Pro were selected for further analysis. Genomic DNA was isolated and the T-DNA insertion site was mapped in R265 mutants by using a PCR based Vectorette system (Sigma, Woodlands, TX). Genomic sequence flanking the insertion site was subsequently obtained by sequencing the PCR product. BLAST analysis using R-265 database at the Broad institute (http://www.broadinstitute.org/annotation/genome/cryptococcus_neoformans_b/MultiHome.html) was performed to reveal the loci containing T-DNA insert.

### Gene deletion and complementation

The primers used in this study are listed in S2 Table. Genes of interest were disrupted via homologous recombination by biolistic transformation [26,28]. Disruption constructs were created using the overlapping PCR technique by linking the 5’ and 3’ flanking region of the gene of interest to selectable

markers *NEO* (G418 resistance), *NAT* (nourseothricin resistance) or *HYG* (hygromycin resistance) [29]. Homologous integrations were confirmed by PCR and Southern hybridization (data not shown). The deletants were complemented by homologous integration using biolistic transformation. *CgDAO2* was reconstituted in *Cgdao1ΔCgdao2ΔCgdao3Δ* triple deletant with the *BLE* (phleomycin resistance) marker.

To overexpress the *DAO* genes, the promoter of *GPD* (Glyceraldehyde-3-phosphate dehydrogenase) as well as the coding and 3’ untranslated region of each *DAO* gene were PCR amplified along with the *HYG* cassette. The PCR products were cloned into *Bam*HI/*Apa*I site of pCR2.1 using the Clontech In-Fusion HD Cloning Plus kit. After confirmation of the construct by DNA sequencing, the linearized DNA fragment was transformed into indicated strains.

For the promoter and gene swap experiment, the *CnDAO2* and *CgDAO2* promoters, which contained 867 bp and 827 bp upstream from the first ATG of the coding region, respectively were PCR amplified. To provide the flanking region for homologous integration, the truncated promoter region (*R2(f)* an *H2(f)*) was PCR amplified. The final construct was obtained by joining the PCR fragments using the Clontech In-Fusion HD Cloning Plus kit. After confirmation of the construct by DNA sequencing, the linearized DNA fragment was transformed into G418 resistant strains of *Cgdao2Δ* or *Cndao2Δ*. The hygromycin resistant transformants were tested for G418 sensitivity. The G418 sensitive transformants, which indicated that homologous integration had occurred at the deleted *dao* locus, were selected and the integration event was confirmed by Southern blot analysis.

### Preparation and analysis of RNA

For northern blot analysis, log phase YPD grown cells were washed and transferred to YNB medium containing 10 mM of the different D-amino acids or ammonium sulfate. RNA was extracted from yeast cells using Trizol (Invitrogen, Carlsbad, CA). ^32^P-labelled DNA probes for northern blots were synthesized from PCR products generated using gene specific primer pairs (S2 Table). The probe for each *DAO* gene did not cross hybridize amongst each other. For quantitative northern analysis, blots were hybridized with the indicated probe, stripped and hybridized with an *ACTIN* probe. The blot was exposed to Phosphorimager Screen and quantified with ImageQuant (Molecular Dynamics). Signal of each *DAO* gene was normalized to that of the *ACTIN* gene and expressed as the relative amount to H99 or R265. For quantitative RT-PCR, log phase YPD grown cells were washed, transferred to YNB medium and grown for 3 h. Cells were washed and transferred to YNB medium containing 10 mM of the different D-amino acids or ammonium sulfate for 2 h. RNA isolation and quantitative RT-PCR was performed as described [26]. The PCR efficiency and CT determination was performed using the algorithm as described [30]. Data were normalized with *ACTIN1* levels and expressed as the amount relative to RNA levels of H99 or R265 grown in ammonium sulfate.

### Virulence studies

The animal experiments were carried out with the approval (#A4149-01) and oversight of the Animal Care and Use Committee of the National Institute of Allergy and Infectious Diseases, National Institutes of Health, USA. Infected mice were monitored daily for signs of lethargy, ruffling, and abnormal ambulation. If mice displayed any one of these criteria, they were then monitored twice daily. Mice were sacrificed by gas cylinder CO2 when they became moribund and that day post infection was considered the survival endpoint. Female BALB/c mice (6–8 weeks old) were injected via the lateral tail vein with 0.2 ml suspension of the indicated yeast strains (2.5 x 10^5^ cells/ml in 0.9% NaCl) [31]. Alternatively, BALB/c mice were inoculated with 5,000 yeast cells via intrapharyngeal aspiration [31,32]. Kaplan-Meier analysis of survival was performed using JMP software for Macintosh (SAS Institute, Cary, NC).

### Recombinant protein production

PCR was used to amplify each *DAO* from *C*. *gattii* and *R*. *toruloides* cDNA. The primers introduced *Xmn*I and *Bam*HI restriction enzyme sequences at the ends of the *DAO* genes to clone into the pMAL-C5X vector (New England Biolabs, Ipswich, MA) cloning site using the Clontech In-Fusion HD Cloning Plus kit (Clontech, Mountain View, CA). The cloned *DAO* gene was inserted downstream of MBP so that the recombinant MBP-Dao proteins could be purified by affinity chromatography. Protein extracts were obtained according to the manufacturer’s suggestions. The horseradish peroxidase/o-dianisidine Dao detection assay was used as previously described [33]. Briefly, crude protein extract was added to a reaction mix containing 50 mM KPO_4_, 0.86 mM o-dianisidine, 100 units horseradish peroxidase, 1.25 uM flavin adenine dinucleotide. After the mixture was heated to 30°C, 100 mM of amino acid was added and the reaction was allowed to progress for 5 minutes. The reaction was stopped by addition of H_2_SO_4_ and the activity was assayed by measuring color changes on a spectrophotometer at OD_540_. The enzyme activity was quantified by comparison to the standard curve generated by pure H_2_O_2_.

## Results and Discussion

### H99 and R265 differ in utilization of D- or L-amino acids as the sole nitrogen source

Although it has been recognized that *C*. *gattii* strains can utilize several D-amino acids more efficiently than *C*. *neoformans*, a comprehensive comparison in the utilization of the common D-amino acids as the sole nitrogen source has not been carried out. We examined the growth capability of H99 and R265 in each of the common D and L-amino acids as the sole nitrogen source. The growth rate of each strain was determined by monitoring OD_600_ at several time points for 24 hours. We found that glycine and ammonium sulfate were good nitrogen sources for both R265 and H99 (Table 1). In general, both H99 and R265 grew better in L-amino acids than in D-amino acids. However, R265 grew better than H99 in most D-amino acids. D-Asn was the only D-amino acid that supported better growth of H99 than R265. D-Gln was the only D-amino acid that served as a good nitrogen source for R265. Three D- and L-amino acids (Cys, Thr, and Tyr), one L-amino acid (L-His) and one D-amino acid (D-Ile) were poor nitrogen sources for either of R265 and H99. These growth patterns delineate the differences between H99 and R265 in utilizing the common D- or L-amino acids as the sole nitrogen source.

**Table 1:** The ability of H99 and R265 to grow in various amino acids as a sole nitrogen source.

	D-amino acid				L-amino acid			
	H99		R265		H99		R265	
	OD_600_ ^a^	ranking^b^	OD_600_	ranking	OD_600_	ranking	OD_600_	ranking
Ala	0.4	+	2.9	++	4.1	+++	5.4	+++
Arg	0.3	+	0.8	+	5.3	+++	6.4	+++
Asn	3.0	+++	1.5	++	6.1	+++	6.4	+++
Asp	0.6	+	1.5	++	6.6	+++	7.6	+++
Cys	0.6	+	0.8	+	0.6	+	0.6	+
Gln	3.2	+++	5.3	+++	4.9	+++	5.2	+++
Glu	0.2	+	1.3	++	0.6	+	3.5	+++
His	0.5	+	1.2	++	0.6	+	0.7	+
Ile	0.5	+	0.9	+	1.2	++	1.0	++
leu	0.5	+	1.6	++	1.6	++	3.2	+++
Lys	0.5	+	1.1	++	3.1	+++	3.1	+++
Met	0.5	+	2.3	++	4.0	+++	1.4	++
Phe	0.5	+	2.5	++	3.3	+++	1.3	++
Pro	0.3	+	2.1	++	4.6	+++	5.4	+++
Ser	0.5	+	2.9	++	2.7	++	5.0	+++
Thr	0.5	+	0.9	+	0.8	+	0.8	+
Trp	0.5	+	1.3	++	3.1	+++	0.6	+
Tyr	0.4	+	0.3	+	0.1	+	0.1	+
Val	0.4	+	1.1	++	0.7	+	1.5	++
Gly					4.4	+++	5.7	+++
NH_4_SO_4_					4.0	+++	4.2	+++

^a.^ Log phase cells were diluted to OD_600_ = 0.1 as starting culture. Indicated amino acid was used as a sole nitrogen source in the liquid YNB media. The culture was incubated at 30°C and the OD_600_ was monitored at several time points for 24h. Only the data from the 24h point are shown. The experiments were repeated twice and the representative data are shown.

^b.^ For comparison, we arbitrary classified the relative growth into three levels: +++ = good N- source, OD_600_ ≥ 4.0; ++ = moderate N-source, 1.0 < OD_600_ < 4.0; + = poor N-source, OD_600_ < 1.0.

### Isolation of R265 mutants that grow poorly on D-Pro or D-Ala as the sole nitrogen source

D-Pro and D-Ala are commonly used to distinguish *C*. *gattii* from *C*. *neoformans* strains [15,17,18]. To characterize the mechanism of D-amino acid metabolism in *C*. *gattii*, a T-DNA insertional library of R265 containing about 20,000 individual clones was first constructed. We then screened for mutants that grew poorly on D-Pro or D-Ala but grew normally on L-Pro or L-Ala. Three such mutants were isolated and the genomic location of the T-DNA insertion in each mutant was determined. Two of the mutants had a T-DNA insertion at either the coding region of CNBG_4524 or the flanking region of CNBG_2060, which were annotated by the Broad Institute as myosin-1 and a hypothetical protein, respectively. In spite of several attempts, we were unable to identify the insertion site of the third mutant. Deletion of the CNBG_4524 gene encoding the putative myosin-1 resulted in a growth defect only on D-Pro but not on D-Ala. We, therefore, focused our studies on CNBG_2060. Sequence analysis indicated that CNBG_2060 contained a Glycine/D-amino acid oxidase domain (COG0665) and we designated this gene as *DAO2* because a putative D-aspartate oxidase gene had been annotated at the Broad Institute (see below). For convenience, we specified the *DAO2* gene from R265 as *CgDAO2* to distinguish it from the orthologous gene of H99 (*CnDAO2*). A BLAST search of the R265 genome using *CgDAO2* as a query sequence revealed the presence of two putative *DAO* paralogs, CNBG_1742 and CNBG_4227, which had been annotated as D-aspartate oxidase and a hypothetical protein respectively by the Broad Institute. We designated CNBG_1742 and CNBG_4227 in R265 as *CgDAO1* and *CgDAO3*, respectively. The sequences of three *DAO* genes from R265 were used as queries to search for the orthologs in the H99 genome. Three orthologs, CNAG_05802, CNAG_03562, and CNAG_02532 were identified and were designated as *CnDAO1*, *CnDAO2* and *CnDAO3*, respectively. S1 Fig shows the protein sequence alignment of these Daos.

### *CgDAO2* is the major *DAO* in R265

**Fig 1:**
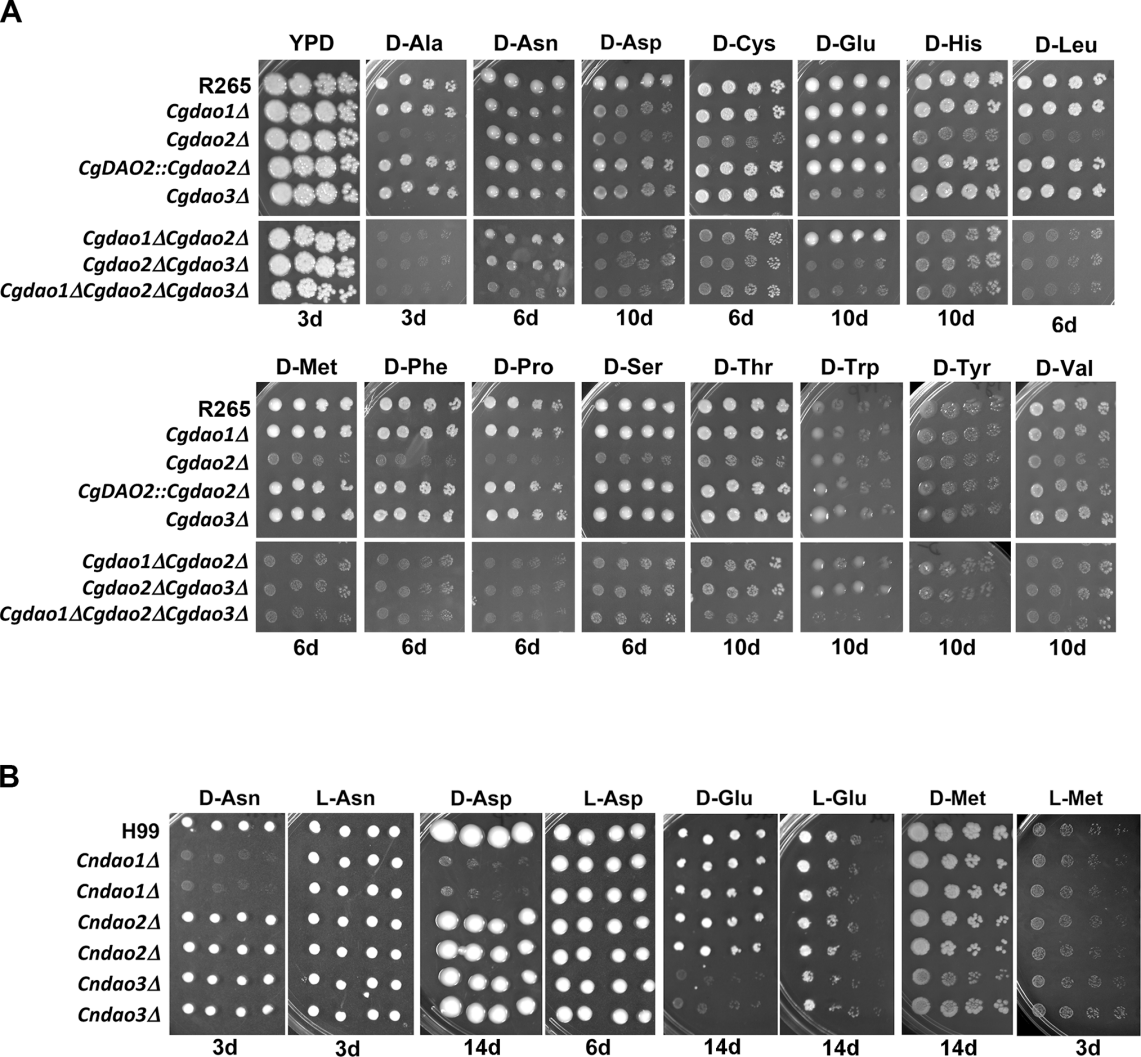
*DAO* genes are important for growth on D-amino acids. (A) Phenotype of R265 *daoΔ* mutants. (B) *CnDAO1* and *CnDAO3* play different roles for growth on D-amino acids. Three-fold serial dilutions of each indicated strain were spotted on indicated medium and incubated at 30°C. The strains used in the experiments are given on the left. Two independent deletants of each *DAO* gene from H99 were assayed. Pictures were taken after incubation for 3 days, 6 days, 10 days or 14 days as indicated. The experiments were repeated twice and representative figures are shown.

Oxidative deamination of D-amino acids by Dao produces ammonia which can be used as the sole nitrogen source for growth. We deleted each of the three *CgDAO* genes in R265 and examined its effect on growth on all common D-amino acids. Deletion of individual *CgDAO1*, *CgDAO2* or, *CgDAO3* genes did not affect growth on any of the common L-amino acids (Table 2) indicating that *DAO* genes are not required for L-amino acids utilization. Deletion of *CgDAO2* resulted in retarded growth on 11 different D-amino acids and reconstitution of *CgDAO2* restored the growth on those D-amino acids (Fig 1A and Table 2) suggesting that *CgDAO2* is the major *DAO* gene required for normal growth of R265 on those 11 D-amino acids. Deletion of *CgDAO3* in R265 markedly reduced the growth on D-Glu and slightly affected the growth on D-D-Asp compared to the wild-type. Growth of *Cgdao1Δ* was also slightly affected in D-Asp. Therefore, D-Asp was the only D-amino acid with which all three single *dao* deletants showed a phenotype of reduced growth. Although deletion of each *CgDAO* gene separately did not clearly affect the growth of R265 on D-Asn, D-Trp or D-Tyr, the triple *CgDAO* gene deletion resulted in severe growth retardation on those three D-amino acids. Interestingly, all R265 *dao* single deletants grew well on D-Arg, D-Gln, D-Ile and D-Lys (Table 2). These amino acids, except for D-Ile, all contain a side-chain amine group that could be used as a nitrogen source. These observations indicate that *DAO* genes are important for growth of R265 on all common D-amino acids that lack the side-chain amine group except for D-Ile. It is possible that additional unidentified D-amino acid oxidases or other enzyme(s) in R265 may convert D-Ile to a useable nitrogen source.

**Table 2:** Summary of growth phenotype associated with deletion of *DAO* genes.

D-amino acid	Deletant showing growth defect on D-amino acid^a^
Ala	*Cgdao2*
Arg	*-* ^*b*^
Asn	*Triple* ^*e*^, *Cndao1*
Asp	*Cgdao1* ^*c*^, *Cgdao2* ^*c*^, *Cgdao3* ^*c*^, *Cndao1*
Cys	*Cgdao2*
Gln	*-* ^*b*^
Glu	*Cgdao3*, *Cndao3*
Gly	NDL^*d*^
His	*Cgdao2*
Ile	*-* ^*b*^
Leu	*Cgdao2*
Lys	*-* ^*b*^
Met	*Cgdao2*, *Cndao3* ^*c*^
Phe	*Cgdao2*
Pro	*Cgdao2*
Ser	*Cgdao2*
Thr	*Cgdao2*
Trp	*Triple* ^*e*^
Tyr	*Triple* ^*e*^
Val	*Cgdao2* ^*c*^

^a^ All *dao* mutants of R265 and H99 grew normally on L-amino acids.

^b^ No phenotype.

^c^ Growth was only slightly reduced compared to the wild-type.

^d^ No D, L-isomers.

^e^ Triple deletant of R265 displayed growth defect on D-amino acid.

### *CnDAO1* and *CnDAO3* play different roles in D-amino acids utilization in H99

Although growth of H99 was ignorable compared to R265 on most D-amino acids (Table 1), H99 grew visibly on certain D-amino acids. Therefore, the role of each *DAO* gene in H99 was also investigated. *CnDAO1* was important for the utilization of D-Asn and D-Asp since deletion of *CnDAO1* reduced the growth of H99 on these D-amino acids (Fig 1B). The growth of *Cndao3Δ* was undetectable on D-Glu and was slightly reduced on D-Met compared to the wild type H99 strain. Deletion of *CnDAO1* or *CnDAO3* did not retard the growth on any of the other D-amino acids. Furthermore, *Cndao2Δ* behaved as the wild-type H99 on every D-amino acid indicating that unlike *CgDAO2* in R265, *CnDAO2* was not important for the growth of H99 on any D-amino acid.

### *DAO3* contributes to adaptation towards D-Glu induced toxicity

**Fig 2:**
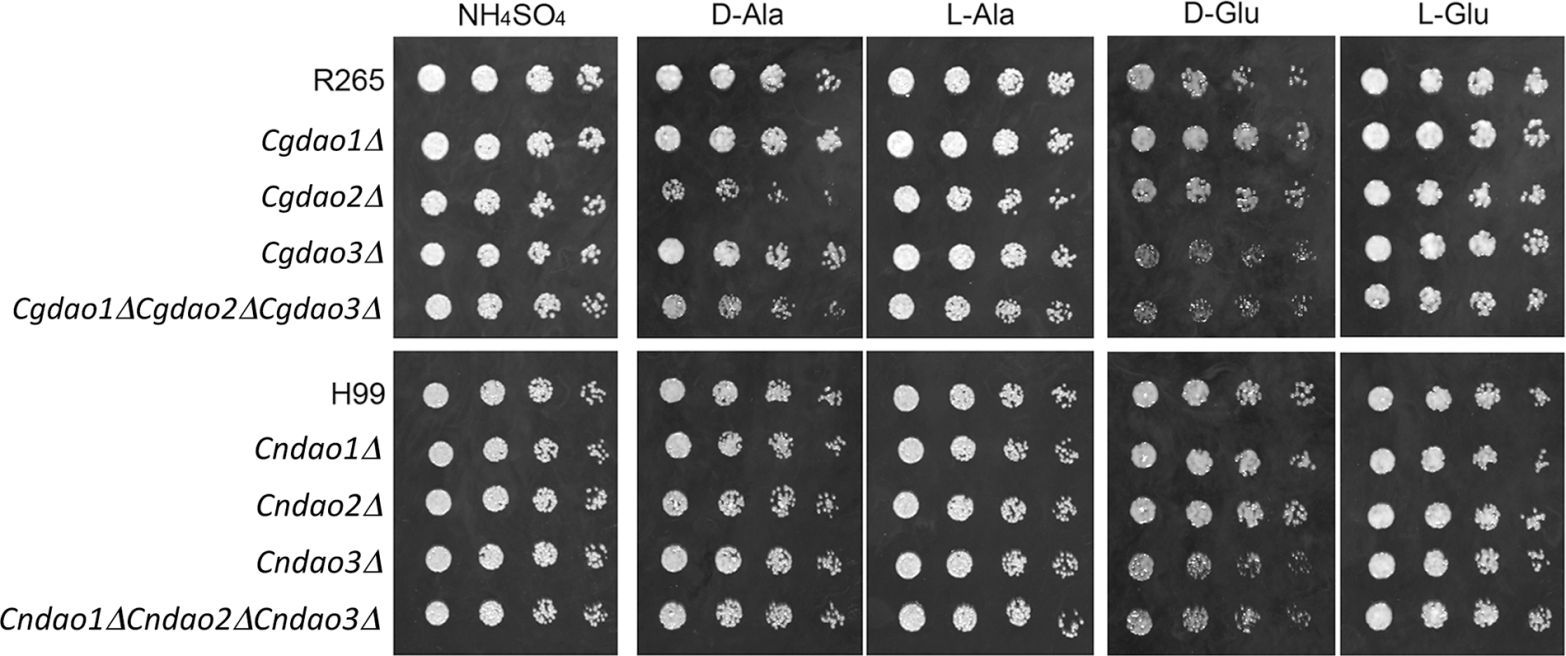
*DAO* genes are important for detoxification of D-amino acids. Three-fold serial dilutions of each strain were spotted onto YNB medium containing 2% glucose and 10 mM ammonium sulfate supplemented with or without 100 mM D- or L-amino acids. Plates were incubated at 30°C for 2 days and photographed. The strains used in the experiments are given on the left.

D-amino acids have been reported to be toxic to many microorganisms including fungi and bacteria [34–37]. We evaluated the toxicity of six representative amino acids including D-Pro and D-Ala which poorly supported the growth of *Cgdao2Δ*, D-Trp which affected growth only on the triple deletant, as well as D-Asn, D-Asp and D-Glu, which failed to support growth of *Cndao1Δ* or *Cndao3Δ* mutant (Fig 1). The strains were spotted on defined agar media containing 2% glucose and 10 mM ammonium sulfate supplemented with or without 100 mM of the selected D- or L-amino acid. Growth of H99 was not affected by the presence of six D-amino acids tested or their L-enantiomers (Fig 2 and S2 Fig). In contrast, addition of D-Glu or D-Ala slightly reduced the growth of wild-type R265. Furthermore, growth of *Cndao3Δ* was slightly reduced in the presence of 100 mM D-Glu whereas growth of *Cgdao2Δ* on D-Ala and *Cgdao3Δ* on D-Glu was slower compared to the R265 strain (Fig 2). Triple and single deletants of *DAO* behaved similarly suggesting no additive effects from the deletion of more than one *CgDAO* gene. Therefore, both *CnDAO3* and *CgDAO3* influenced growth and detoxification of D-Glu in H99 and R265 respectively. In contrast, only *CgDAO2* but not *CnDAO2* influenced growth and detoxification of D-Ala.

### Expression patterns of *DAO* genes in eight different D-amino acids

**Fig 3:**
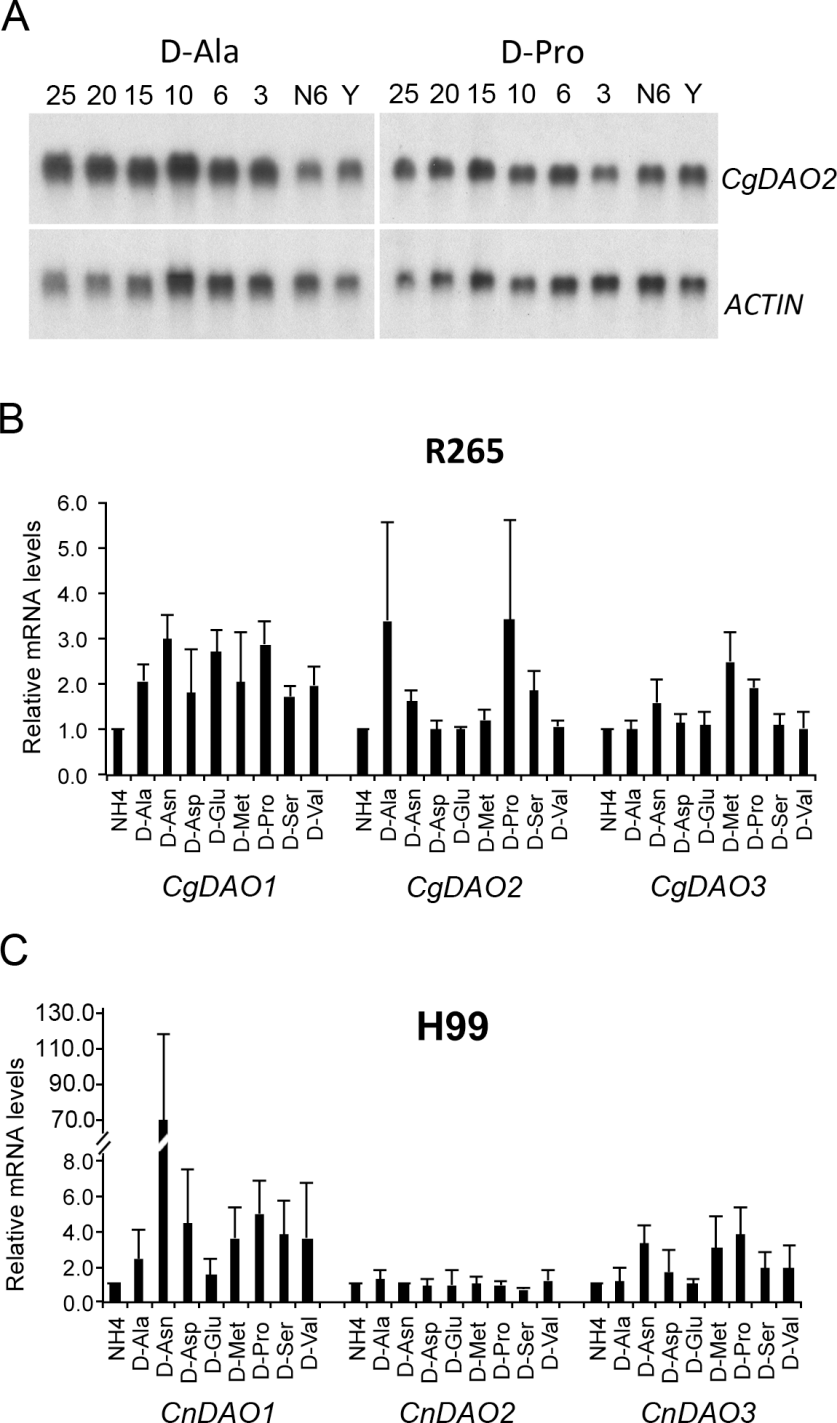
Expression of *DAO* genes is induced by certain D-amino acids. (A) *CgDAO2* expression increases in the presence of D-Ala or D-Pro. YPD grown cells (Y) were washed and transferred to YNB medium containing 10 mM D-Ala, D-Pro or ammonium sulfate (N) for the indicated hours. Total RNA (5 μg) was subjected to northern blot analysis using a *CgDAO2* probe. Actin served as loading control. (B and C) Expression profiles of R265 and H99 *DAO* genes in various D-amino acids. YPD grown cells were washed and transferred to YNB medium containing 10mM of the indicated D-amino acids or ammonium sulfate for 2 h. Northern blots were hybridized with the indicated *DAO* probes. Signals of each *DAO* gene were normalized to that of the *ACTIN* gene and expressed as the relative amount to R265 or H99 RNA from the ammonium sulfate grown cultures. The experiments were repeated three times and the error bar represents standard deviation.

In various fungi, the expression of *DAO* is induced in the growth media where a D-amino acid is the sole nitrogen source [38–40]. Since growth of R265 on D-Ala and D-Pro required *CgDAO2*, we examined the expression patterns of *CgDAO2* in media containing D-Ala or D-Pro as the sole nitrogen source. The expression of *CgDAO2* was detectable in the rich medium YPD, and its expression did not change substantially when the cells were shifted from YPD medium to defined medium containing ammonium sulfate as the sole nitrogen source for 6 h (Fig 3A). However, when R265 was shifted from YPD to D-Ala as the sole nitrogen source, expression of *CgDAO2* increased at 3 h after transfer and maintained the increased levels for at least 25 hours. Similarly, expression of *CgDAO2* increased slightly at 6 h after shifting from YPD to D-Pro and maintained the increased levels for at least for 25 hours. Therefore, *CgDAO2* is required for the growth on D-Ala and D-Pro and its expression levels increase in the presence of these two D-amino acids.

We expanded the gene expression analysis of each *DAO* in both R265 and H99 using northern blot analysis. Eight different D-amino acids were selected based on growth phenotypes; five D-amino acids in which *Cgdao2Δ* showed a growth deficiency and three D-amino acids in which *Cndao1Δ* or *Cndao3Δ* grew poorly. Fig 3B shows that in R265, the expression levels of *CgDAO2* increased more than two-fold only in the cells grown on D-Ala or D-Pro while no such change occurred in those grown in other D-amino acids. Although deletion of *CgDAO1*, *CgDAO2* and *CgDAO3* each affected the growth of R265 on D-Asp slightly (Fig 1A), none of these genes showed a greater than two-fold increase of the expression levels in D-Asp grown cells. Furthermore, expression levels of *CgDAO1* increased more than two-fold in six out of the eight tested D-amino acids while expression levels of *CgDAO3* increased more than two-fold in one (D-Met) out of the eight tested D-amino acids.

In H99, expression of *CnDAO1* was highly elevated in media containing D-Asn (Fig 3C). Additionally, the expression levels of *CnDAO1* in H99 were elevated greater than 2-fold in seven out of the eight tested D-amino acids even though deletion of *CnDAO1* only showed growth retardation on D-Asp and D-Asn (Fig 1B and Table 2). Similarly, the expression levels of *CnDAO3* increased more than two-fold in D-Asn, D-Met, and D-Pro while deletion of *CnDAO3* did not affect the growth of H99 in those D-amino acids. In contrast, the expression of *CnDAO3* did not increase in D-Glu even though *CnDAO3* was important for the growth on D-Glu or detoxification of D-Glu. Lastly, the expression of *CnDAO2* did not change more than two-fold in all eight tested D-amino acids. Since deletion of *CnDAO2* had no phenotype in all the tested conditions and expression of *CnDAO2* was not inducible by any of the tested D-amino acids, the function of *CnDAO2* is not clear for D-amino acid utilization in H99. Taken together, results of the expression profile analysis indicate that the relationship between the growth phenotype of each *dao* mutant and the expression levels of the corresponding *DAO* gene in both H99 and R265 is not well-defined. It is known that Gat1 belongs to a conserved family of zinc finger containing transcriptional regulators which activate the transcription of nitrogen catabolite repression sensitive genes when preferred nitrogen sources are absent or limiting [15,41]. It is possible that *GAT1* may also control the expression of *DAO* genes in different D-amino acids. However, no clear relationship was observed between the expression profile of each *DAO* genes and deletion of *GAT1* in H99 or R265. It is possible that other factors may mitigate the expression levels of specific *DAO* genes in specific D-amino acids.

### Certain *DAO* genes are functionally interchangeable when overexpressed

Since the results of phenotypic analysis suggested that each *DAO* gene functions differently in D-amino acid utilization, we next examined if each *DAO* gene could functionally substitute other *DAO* genes by overexpressing individual *DAO* gene under control of the strong *GPD* promoter. D-amino acids that affected the growth of single *dao-*deletion mutants were divided into three groups to test the effect of *DAO* gene overexpression. The first group included 10 D-amino acids in which *Cgdao2Δ* grew poorly (Table 2). D-Ala, D-Pro and D-Met were selected as the representatives of this group for the test. D-Glu was the second group since *Cgdao3Δ* and *Cndao3Δ* strains both failed to use D-Glu as the nitrogen source. The last group included D-Asp in which the growth of *Cgdao1Δ*, *Cgdao2Δ*, *Cgdao3Δ* and *Cndao1Δ* was retarded.

**Fig 4:**
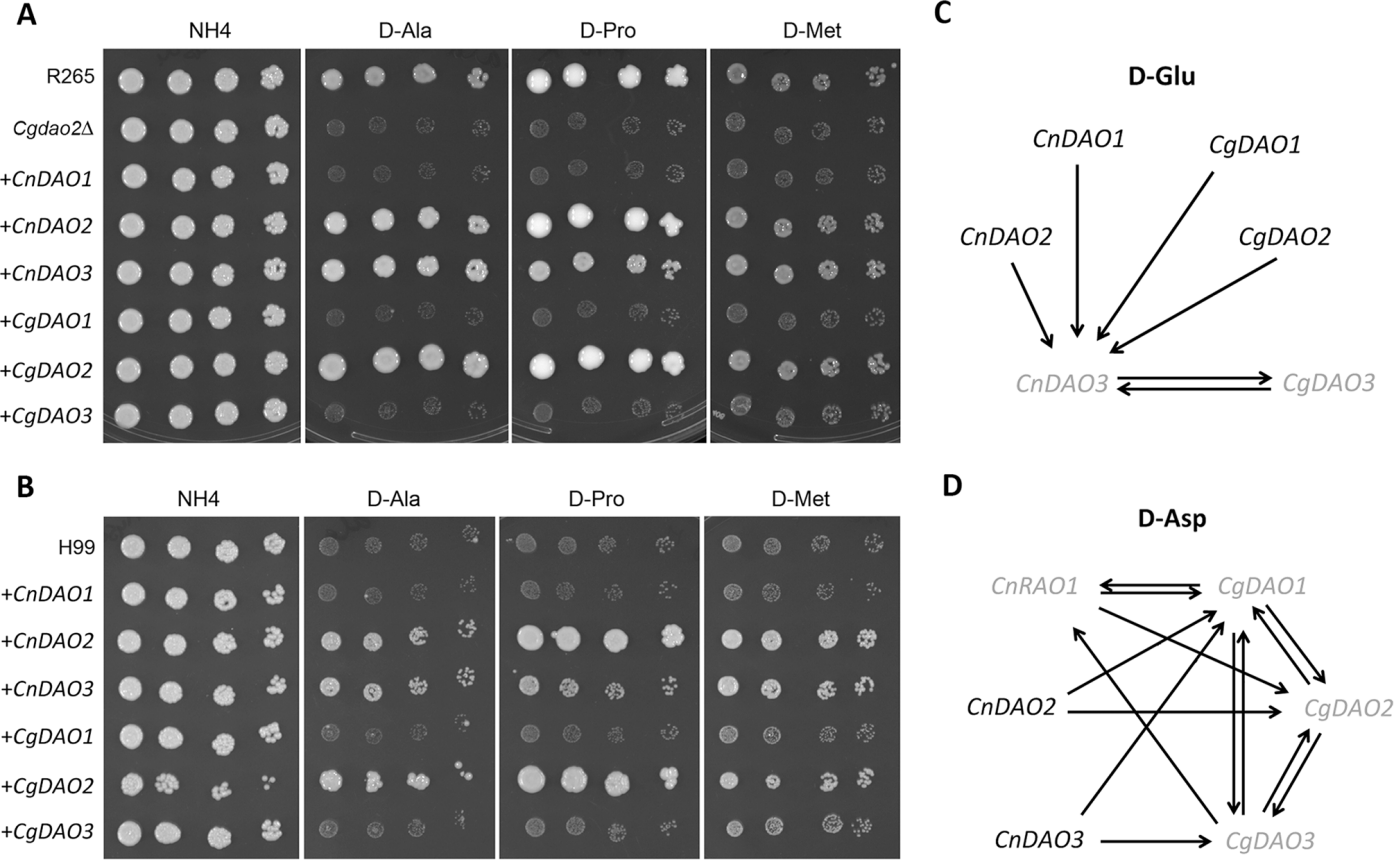
Some *DAO* genes are functionally interchangeable when overexpressed. (A) Overexpression of *CnDAO2* and *CnDAO3* complements the growth deficiency of *Cgdao2Δ* on three D-amino acids. Strain *Cgdao2Δ* was transformed with the overexpression construct of the indicated *DAO* gene. The resulting strains were spotted onto YNB medium containing ammonium sulfate or the indicated D-amino acids and incubated at 30°C. Pictures were taken after 3 days (NH_4_) or 5 days (D-Ala, D-Pro and D-Met) of incubation. (B) H99 grows robustly on three D-amino acids when *DAO* genes are overexpressed. H99 was transformed with the overexpression construct of indicated *DAO* gene. Spot assays were performed as in (A). (C) Overexpression of every *DAO* gene complements the growth deficiency of *Cndao3Δ*. *Cndao3Δ* and *Cgdao3Δ* were transformed with each *DAO* overexpression constructs. The resulting transformants were tested for the ability to grow on D-Glu and the results are summarized. Lighted color letters indicate the original mutants that grow poorly on D-Glu. Dark color letters indicate the original mutants that have no phenotype on D-Glu. Arrow line indicates that overexpression of indicated *DAO* genes complement the mutant phenotype on D-Glu. (D) Complementation of the growth deficiency of *dao* mutants on D-Asp by overexpressing *DAO* genes. The *dao* mutants showing growth defect on D-Asp were transformed with *DAO* overexpression constructs. The resulting transformants were tested for the ability to grow on D-D-Asp. Symbols used in the figure are the same as in (C).

In the first group, D-Ala, D-Pro and D-Met, only *CgDAO2* but not *CgDAO1* or *CgDAO3* was able to complement the growth defect of *Cgdao2Δ* (Fig 4A). In contrast, overexpression of *CnDAO2* and *CnDAO3* but not *CnDAO1* complemented the growth deficiency of *Cgdao2Δ* on D-Ala, D-Pro and D-Met suggesting that CnDao2 and CnDao3 could oxidize those three D-amino acids when overexpressed in R265.

Surprisingly, the H99 strain, which grew poorly on D-Ala, D-Pro and D-Met as the sole nitrogen source, grew robustly on these D-amino acids when *CgDAO2* was overexpressed in H99 (Fig 4B). Furthermore, overexpression of *CnDAO2* and *CnDAO3* also enabled H99 to utilize those three D-amino acids. These results suggest that CnDao2 and CnDao3 can convert D-Ala, D-Pro and D-Met into a usable nitrogen source when overexpressed in both H99 and R265. These data also suggest that CnDao1, CgDao1 and CgDao3 lack the ability to oxidize the D-amino acids belonging to the first group.

In the second group, D-Glu, overexpression of each *DAO* gene from both H99 and R265 enabled growth of *Cndao3Δ* on D-Glu and the results are summarized in Fig 4C. These data suggest that every *DAO* gene can functionally substitute for *CnDAO3* in H99 and enables the strain to utilize D-Glu as the nitrogen source. In contrast, growth of *Cgdao3Δ* on D-Glu was restored only by over expression of *CnDAO3* and not by any other *DAO* gene. As mentioned above, growth of R265 and not H99 was affected by the presence of 100 mM D-Glu, thus it is possible that H99 contains additional factors related to D-Glu utilization/tolerance compared to R265. The detailed mechanisms of these differences are yet to be elucidated.

In the third group, D-Asp, the effect of overexpression of each *DAO* gene was examined in *Cgdao1Δ*, *Cgdao2Δ*, *Cgdao3Δ and Cndao1Δ* strains and the results are summarized in Fig 4D. Overexpression of each *DAO* gene from either R265 or H99 functionally complemented the growth defect of at least two R265 *dao* mutants on D-Asp. These data suggest that the Daos from H99 and R265 can catabolize D-Asp when overexpressed in R265. In contrast, *CnDAO2* and *CnDAO3* were not able to complement the growth defect of *Cndao1Δ* on D-Asp. In addition, only *CgDAO1* and *CgDAO3* but not *CgDAO2* were able to complement the growth defect of *Cndao1Δ*. Taken together, our results suggest that every Dao of R265 and H99 has ability to convert all the D-amino acids belonging to the second and third groups into a useable nitrogen source whereas only CgDao2, CnDao2, and CnDao3 have the ability to catabolize the D-amino acids in the first group.

### Dao substrate specificity

Our genetic studies suggested the substrate specificity of each Dao. We attempted to biochemically determine the substrate specificity of each Dao using *dao* mutants of H99 and R265. However, Dao enzymatic activity was undetectable in crude total protein extracts from H99 and R265 while it could be readily detected from a *Rhodosporidium toruloides* strain known to be a high Dao producer [33,42,43]. Dao activity is known to vary greatly among different organisms [44,45]. It is possible that cryptococcal Daos have very low enzyme activity. To further characterize the Dao protein function, CgDao1, CgDao2 and CgDao3 were individually expressed in *E*. *coli* as a recombinant protein fused to maltose-binding protein (MBP). Recombinant Dao of *R*. *toruloides* (RtDao) produced by the same vector was used as a positive control.

Table 3 shows the substrate specificity of the recombinant Dao proteins. Eleven D-amino acids that affected the growth of *Cgdao1*, *Cgdao2*, or *Cgdao3* deletion mutants were examined. The positive control, RtDao, displayed high enzymatic activity towards most D-amino acids except for D-Asp and D-Phe. In contrast, the enzymatic activity of all three recombinant R265 Dao proteins was markedly lower compared to RtDao although the amounts of each recombinant CgDao and RtDao protein produced in *E*. *coli* were similar (S3 Fig). Low Dao enzyme activity of the recombinant R265 Dao proteins corroborated our inability to detect the Dao enzymatic activity from crude protein extracts of R265.

**Table 3:** Substrate specificity of Dao enzymes.

Protein	RtDao	CgDao1	CgDao2	CgDao3	Concordance with growth phenotypes^a^
D-Ala	41.22	0.040	0.218*	0.043	Yes-*dao2Δ*
D-Asp	0.97	0.916*	0.053	0.049	Yes^b^
D-Glu	73.86	0.049	0.080	0.032	No-*dao3Δ*
D-His	41.26	0.049	0.128*	0.031	Yes-*dao2Δ*
D-Leu	15.84	0.044	0.153*	0.045	Yes-*dao2Δ*
D-Met	27.77	0.050	0.64*	0.040	Yes-*dao2Δ*
D-Phe	3.97	0.046	0.043	0.031	No-*dao2Δ*
D-Pro	39.81	0.054	0.136*	0.041	Yes-*dao2Δ*
D-Ser	31.13	0.062	0.129*	0.044	Yes-*dao2Δ*
D-Thr	17.70	0.040	0.052	0.036	No-*dao2Δ*
D-Val	50.55	0.055	1.009*	0.039	Yes-*dao2Δ*

^a^ Substrate specificity of CgDao enzymes (nM H_2_O_2_/ug crude extract) was compared to the growth phenotypes of *Cgdao* mutants listed in Table 2. “Yes” or “No” designates whether the ability to oxidize D-amino acid is concordant with the growth phenotypes of the specified *CgdaoΔ* mutant. The experiments were repeated twice and the representative data are shown.

^b^ CgDao1 showed the clearest concordant substrate specificity with *Cgdao1Δ* growth phenotype.

* Activity is at least two fold higher than the control activity of CgDao using L-amino acid as substrate.

Despite low activity of the three R265 Dao enzymes, CgDao2 showed the broadest substrate specificity. The patterns of CgDao2 and RtDao substrate specificity were similar except that CgDao2 showed relatively lower activity against D-Glu and D-Thr. We noted that substrate specificity of the recombinant CgDaos did not correlate perfectly with the growth phenotypes of each *Cgdao* mutant (Table 3). For instance, CgDao1 was the only Dao protein which displayed appreciable activity using D-Asp as a substrate and the recombinant CgDao3 showed no appreciable oxidative activity with the substrates tested. Because the recombinant Dao is a MBP fusion protein, it is possible that the MBP fusion caused the observed discrepancy between substrate specificity and growth phenotype. This possibility is supported by the observation that while recombinant RtDao had the highest activity when D-Glu was used as the substrate (Table 3), Dao activity of the native *R*. *toruloides* protein was very low against D-Glu [33,43]. The CgDao2 recombinant protein was purified further by removing the MBP with Factor Xa. However, the MBP cleaved CgDao2 lost its ability to oxidize D-amino acids. Since the enzymatic activity of all R265 recombinant Dao proteins was low and our data suggested that the enzymatic activity of CnDao2 was even weaker than CgDao2 (see below), we have not attempted to express any Dao of H99 in *E*. *coli*. The precise substrate specificity of each Dao may require further biochemical analysis using highly purified native proteins.

### Gene and promoter swap between *CgDAO2* and *CnDAO2*

**Fig 5:**
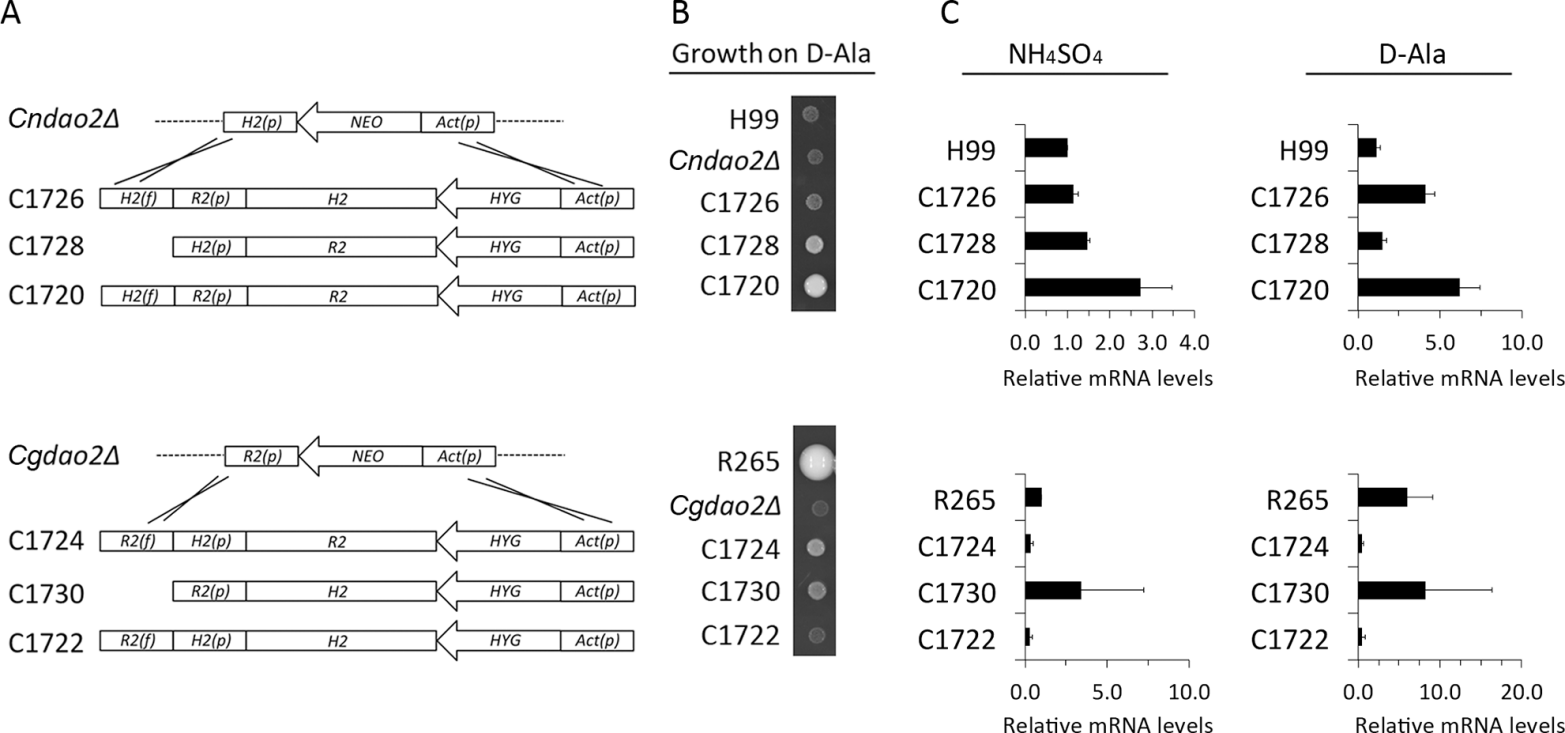
Gene and promoter swap between *CnDAO2* and *CgDAO2*. (A) Diagram of the constructs. *Cndao2Δ* and *Cgdao2Δ* were transformed with the indicated constructs. The names of the resulting strains are listed on the left. Dotted line indicates the chromosomal regions flanking the deleted locus. Crosses indicate the crossing over event at the homologous regions. The symbols *Act(p)* = Actin promoter; *HYG* = hygromycin resistance gene; *NEO* = neomycin resistance gene; *H2* = *CnDAO2* without the promoter; *R2* = *CgDAO2* without the promoter; *H2(f)* and *R2(f)* = flanking region of *CnDAO2* and *CgDAO2* respectively; *H2(p)* and *R2(p)* = promoter of *CnDAO2* and *CgDAO2* respectively. (B) Spot assay of the gene swapped strains. Approximately 600 cells were spotted on D-Ala and the plates were incubated at 30°C for 11 days and photographed. (C) Relative RNA levels. Log phase cells of the indicated strains were transferred to YNB medium containing 10 mM ammonium sulfate or D-Ala for 2 h and the RNA was isolated. The relative mRNA levels were determined by quantitative RT-PCR. Data were normalized with *ACTIN* levels and expressed as the relative RNA levels of H99 (upper panel) or R265 (lower panel) grown in ammonium sulfate. The experiments were repeated three times and the error bars represent the standard deviation of three technical repeats.

*S*ince *CgDAO2* is the major *DAO* gene in R265 and overexpression of *CnDAO2* complemented the *Cgdao2Δ* phenotype, we further explored the function of *DAO2* genes in both species. Since overexpression of *CgDAO2* using the *GPD* promoter enabled the H99 strain to utilize several D-amino acids, we tested if using the native *CgDAO2* promoter could have a similar effect. Insertion of the entire *CgDAO2* gene into the *Cndao2Δ* locus in H99 (Fig 5A upper panel; *R2(p)-R2*; strain C1720) enabled its growth on D-Ala (Fig 5B). This result indicates that a single copy of the *CgDAO2* gene enables H99 to utilize D-Ala. When the *CgDAO2* promoter in C1720 was swapped with that of *CnDAO2*, the resulting strain, C1728 (*H2(p)-R2*), grew better on D-Ala than H99 but weaker than C1720. The transcriptional levels of *CgDAO2* determined by quantitative RT-PCR were higher in C1720 than in C1728 when the cells were grown in ammonium sulfate (Fig 5C). The transcription levels of *CgDAO2* were slightly induced in C1720 by D-Ala but not in C1728. These data suggest that the apparent strength of the *CgDAO2* promoter relative to that of *CnDAO2* may explain the growth difference between C1720 and C1728 on D-Ala.

When the promoter of *CnDAO2* was replaced with that of *CgDAO2*, the resulting strain, C1726 (*R2(p)-H2*), grew slightly better than H99 but poorer than C1728 on D-Ala although *CnDAO2* RNA levels in C1726 were higher than the *CgDAO2* RNA levels in C1728 on D-Ala (Fig 5C). Since the only difference between these strains was at the *CnDAO2* locus, it is possible that the observed growth difference may be due to higher enzymatic activity of CgDao2 in C1728 compared to that of CnDao2 in C1726. The possibility that enzymatic activity of CgDao2 is higher than CnDao2 is supported by the observation that growth of the strains overexpressing *CgDAO2* was more robust than those overexpressing *CnDAO2* in which both overexpression constructs were under the control of the same *GPD* promoter (Fig 4A and 4B).

We have shown that overexpression of *CnDAO2* was able to complement the *Cgdao2Δ* phenotype on D-Ala. To examine if the native *CnDAO2* promoter could provide a similar effect in the *Cgdao2Δ* background, the entire *CnDAO2* gene was inserted into the *Cgdao2Δ* locus (Fig 5A bottom panel). The resulting strain, C1722 (*H2(p)-H2*), failed to restore the growth of *Cgdao2Δ* on D-Ala (Fig 5B). When the *CnDAO2* promoter was replaced by that of *CgDAO2*, the resulting strain, C1730 (*R2(p)-H2*), was able to grow on D-Ala although the growth was considerably weaker than R265. Furthermore, when the coding region of *CnDAO2* was replaced by that of *CgDAO2*, growth of the resulting strain, C1724 (*H2(p)-R2*), on D-Ala was more robust than C1730 although the transcription levels of *CgDAO2* were much lower in C1724 than that of the *CnDAO2* in C1730 (Fig 5C bottom panel). These results support the notion that the enzymatic activity of CgDao2 may be stronger than CnDao2. These data also indicate that although the promoter strength of *CnDAO2* is weaker than *CgDAO2* in R265 background and the expression levels of *CnDAO2* was not affected by the presence of different D-amino acids (Figs 3C and 5C), *CnDAO2* promoter is functional and capable of driving the expression of *CgDAO2* in strain C1724 allowing *Cgdao2Δ* to grow moderately on D-Ala.

### *DAO* genes play a role in virulence of R265 in murine models

**Fig 6:**
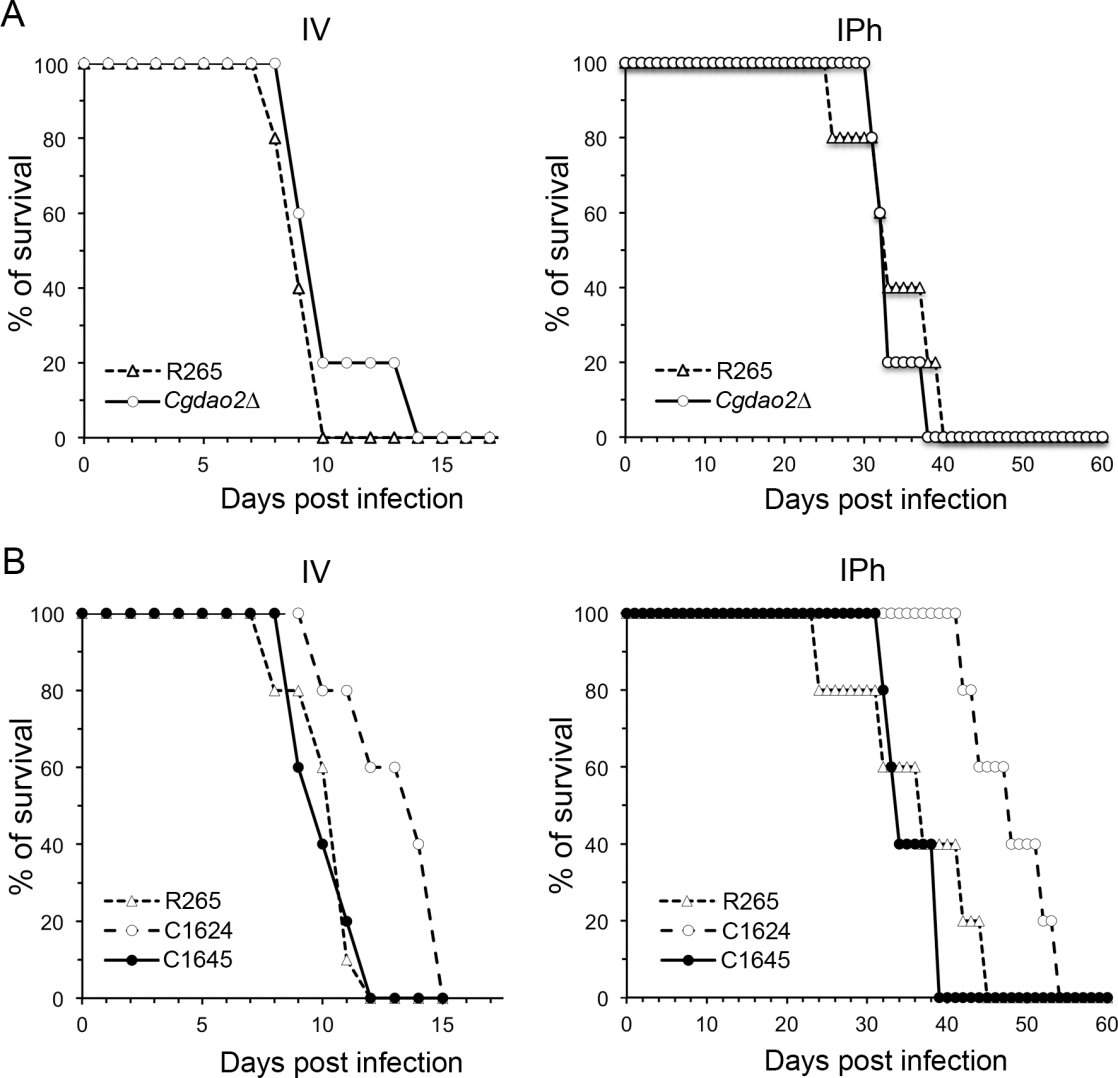
Virulence of the triple *dao* deletant of R265 is reduced. (A and B) BALB/c female mice (5 per group) were infected with the indicated strains either by intravenous injection (IV) or intrapharyngeal aspiration (IPh) and the mortality was monitored. C1624: *Cgdao1ΔCgdao2ΔCgdao3Δ* triple deletant; C1645: *Cgdao1Cgdao3Δ* double deletant (derived from C1624 by reconstituting *CgDAO2*). The experiments were repeated twice and the representative data are shown.

Since *CgDAO2* was the major *DAO* gene in R265, we examined the role of *CgDAO2* in virulence using murine models. Deletion of *CgDAO2* did not affect virulence in mice infected either by intrapharyngeal aspiration (IPh) or intravenous injection (IV) (Fig 6A). Similarly, neither the deletion of *CgDAO1* nor *CgDAO3* affected virulence of R265 (data now shown). Interestingly, virulence of the *Cgdao1ΔCgdao2ΔCgdao3Δ* triple deletant (C1624) was reduced compared to the wild-type in both IPh (*p* = 0.018, log-rank test) and IV (*p* = 0.041, log-rank test) models while complementation of *CgDAO2* in the triple deletant (C1645) restored the virulence (Fig 6B). This suggests that although the individual *DAO* gene is not required for the virulence of R265, the three *CgDAO* genes cooperatively contribute to virulence. On the other hand, the *Cndao1ΔCndao3Δ* double deletant or the *Cndao1ΔCndao2ΔCndao3Δ* triple deletant exhibited virulence similar to H99 suggesting that *DAO* genes in H99 do not play a role in pathogenicity (S4A Fig).

Since there are many other nitrogen sources other than D-amino acids available in the host, the observed modest pathological effect of triple deletion in R265 could be unrelated to their ability to utilize D-amino acids for growth. However, persistently high concentrations of D-Ser have been reported in the mammalian forebrain [21,22]. We suspected that R265 *DAO* genes might play a role in brain tropism. We therefore compared the effectiveness of the R265 brain invasion to that of the three different *dao* mutants. Since R265 cells can efficiently cross the blood-brain barrier and produce severe meningoencephalitis when inoculated intravenously [31], mice were infected via the tail vein and the number of colony forming units in the brain were determined after 3h. No significant difference was found in the brain’s fungal load between the mice infected with the wild-type and those infected with *daoΔ* mutants including the *Cgdao1ΔCgdao2ΔCgdao3Δ* triple deletant (S4B Fig). We also compared the brain fungal load of mice challenged by IPh after 7, 14 and 35 days post infection and found no difference between the mice infected with wild-type and those with the triple deletant (S4C Fig). These observations indicate that the *dao* genes of R265 do not play a role in brain tropism in mice.

Although *DAO* genes and utilization of D-amino acids have been studied in various fungal species [34–36,38–40,42], this is the first study dealing with a comprehensive analysis of each *DAO* paralogous gene function in two genetically close pathogenic fungi. Our interest in comparing the mechanism responsible for D-amino acid utilization by the two etiologic agents of cryptococcosis stems from practical reasons. Utilization of certain D-amino acids as the sole nitrogen source has been used as a biochemical tool to distinguish the two species. We chose a representative strain from each species, H99 for *C*. *neoformans* and R265 for *C*. *gattii*, to determine growth differences in the common D-amino acids. Although three putative D-amino acid oxidases were found in both species, H99 grew poorly in most D-amino acids except for D-Asn and D-Gln. By deleting each *DAO* gene, we have demonstrated that each *DAO* gene functions differently with respect to utilization of D-amino acids in H99 and R265 strains.

Several lines of evidence in combination could explain why H99 cannot grow on various D-amino acids. First, overexpression of *CnDAO2* and *CnDAO3* in H99 enables growth on several D-amino acids suggesting that one of the reasons for the inability of H99 to grow may be due to insufficient expression levels of the *CnDAO* genes. Second, although expression levels of the *DAO2* gene were inducible when the gene was driven by the *CgDAO2* promoter in H99 (C1720 and C1726; Fig 5C), the trend of the expression levels was slightly lower in H99 background than in R265 (C1720 vs. R265 and C1726 vs. C1730). These results suggest that H99 may lack some of the components required for an adequate control of *DAO* gene expression. Lastly, the results of gene and promoter swaps along with overexpression studies further suggest that the enzymatic activity of CnDao2 was lower compared to that of CgDao2.

The major ecological niche of *C*. *gattii* is plants [1] and several D-amino acids are known to be present in higher plants [20]. It is possible that the ability to utilize D-amino acids may have been incorporated into *C*. *gattii* during the evolutionary process. The virulence difference between triple deletants in R265 and H99 highlights the fact that *DAO* has evolved differently in these two species. It is not clear, however, if involvement of *DAO* genes in the pathobiology was a gained function in R265 or a lost function in H99 during evolution.

## Supporting Information

S1 Fig Sequence alignment of Daos from H99, R265 and other fungi. Amino acid sequences of DAOs from AhDao (*Asterotremella humicola*; AB121230), RtDao (*Rhodosporidium toruloides*; P80324), and TvDao (*Trigonopsis variabilis*; Q99042), R265 and H99 were compared by CLUSTER W alignment program.
(TIF)

S2 Fig Toxicity test of DAs. Three-fold serial dilutions of each strain were spotted onto YNB medium containing 2% glucose and 10 mM ammonium sulfate supplemented with or without 100 mM D- or L-amino acids. Plates were incubated at 30°C for 2 days and photographed.
(TIF)

S3 Fig Production of Dao recombinant proteins. (A) *E coli*. crude protein extract was isolated, separated in a 4–12% NuPAGE gel and stained with Comassie Blue stain reagent. Twenty ug of Cgdao2 and RtDao and 30 ug of Cgdao1 and Cgdao3 post-induction samples were loaded in wells. Approximately the same amount of pre-induction sample was loaded in wells. (B) Purity of the Dao recombinant proteins after affinity chromatography. Protein was separated by a 4–12% NuPAGE gel and stained with Comassie Blue reagent. Samples 1, 3, and 5 contain crude extract (10ug) and samples 2, 4, and 6 contain purified sample (2ug). Cgdao3 was not shown due to lack of detectable enzymatic activity.
(TIF)

S4 Fig (A) Virulence H99 dao deletants is the same as the wild-type. BALB/c female mice (5 per group) were injected with various strains either by intravenous injection (IV) or intrapharyngeal aspiration (IPh) and the mortality was monitored. (B) Frequency of brain entrance is not reduced in the *Cgdao* triple deletant. Group of three mice each were infected intravenously with the indicated strains. Brains were isolated 3h post infection and the colony forming units in the brain were determined. Data is expressed as a percentage of the number of the CFU’s in the brain vs. the number of input cells. Bar: mean value of three mice. (C) Fugal burden in the brain of the mice infected by the *Cgdao* triple deletant is the same as the wild-type. Group of three mice each were infected by intrapharyngeal aspiration. Brains were isolated from the infected mice as indicated time and the colony forming units (CFU) per the brain were determined.
(TIF)

S1 Table List of strains relevant to this study. (DOCX)

S2 Table Primers relevant to the study. (DOCX)
