# Supplementary figures and images for: Differences between Cryptococcus neoformans and Cryptococcus gattii in the Molecular Mechanisms Governing Utilization of D-Amino Acids as the Sole Nitrogen Source
Source: PLoS One. 2015 Jul 1;10(7):e0131865. doi: 10.1371/journal.pone.0131865 (PMC4489021; doi:10.1371/journal.pone.0131865)

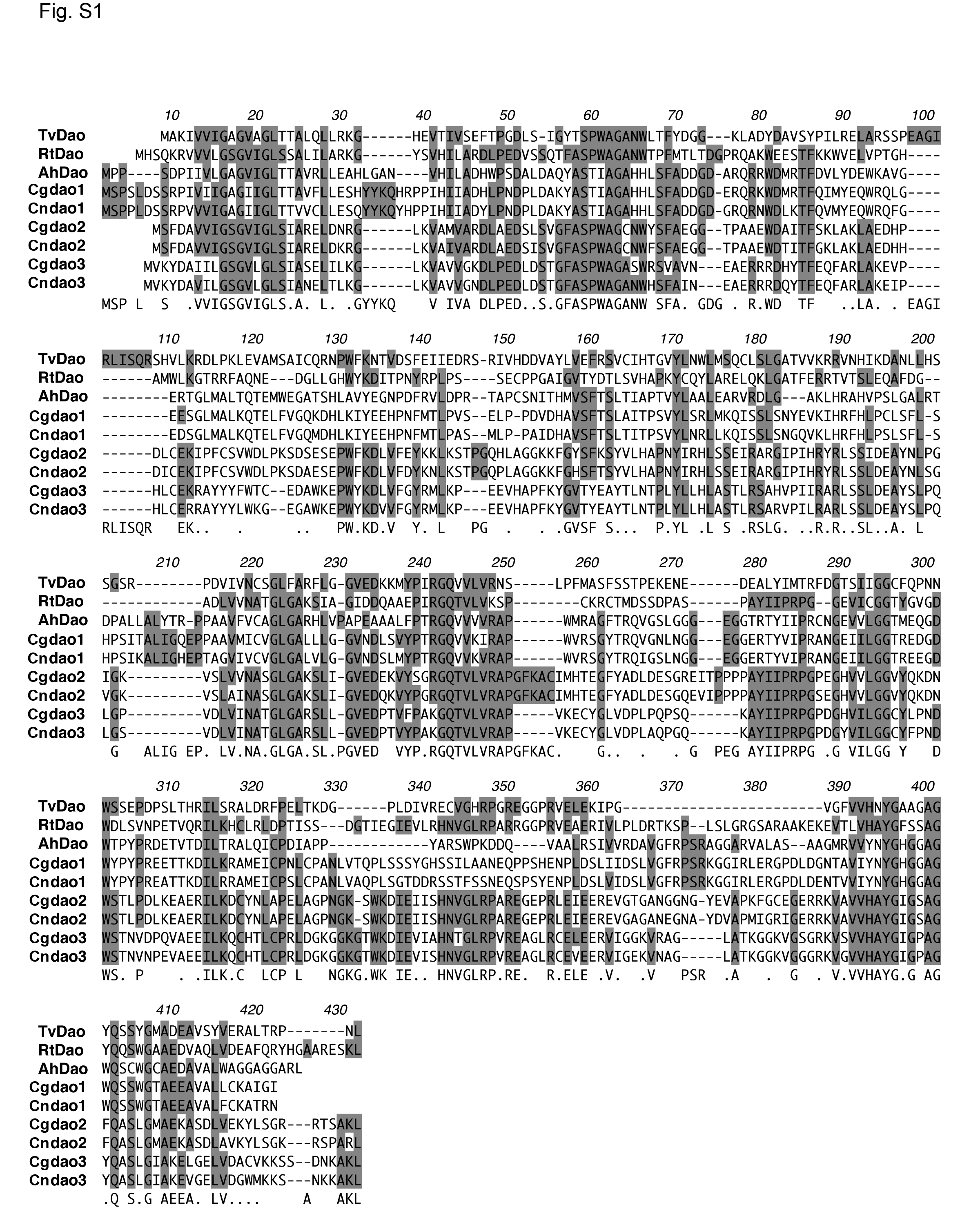

Supplement: S1 Fig — Amino acid sequences of DAOs from AhDao (Asterotremella humicola; AB121230), RtDao (Rhodosporidium toruloides; P80324), and TvDao (Trigonopsis variabilis; Q99042), R265 and H99 were compared by CLUSTER W alignment program. (TIF) [file pone.0131865.s001.tif]

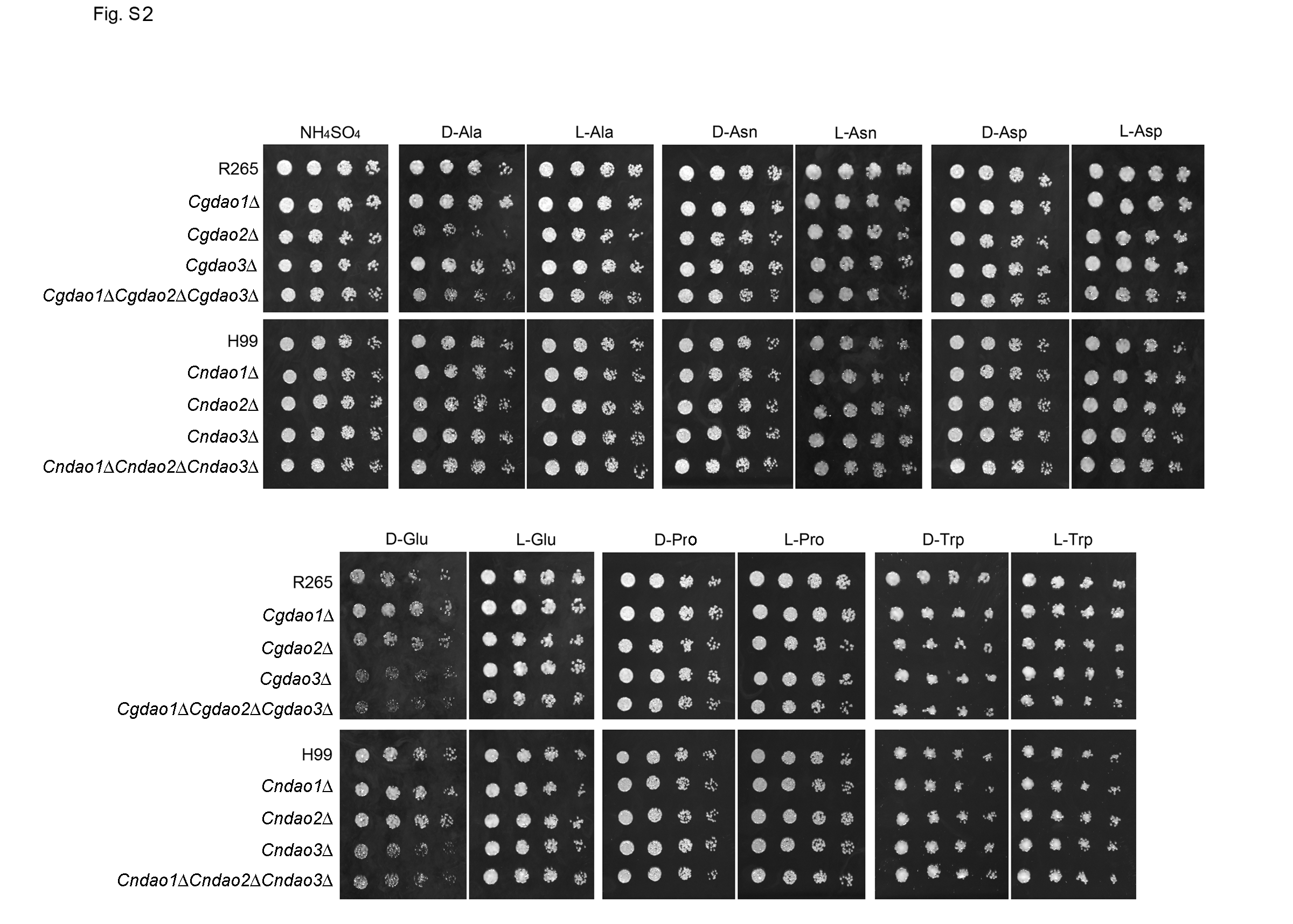

Supplement: S2 Fig — Three-fold serial dilutions of each strain were spotted onto YNB medium containing 2% glucose and 10 mM ammonium sulfate supplemented with or without 100 mM D- or L-amino acids. Plates were incubated at 30°C for 2 days and photographed. (TIF) [file pone.0131865.s002.tif]

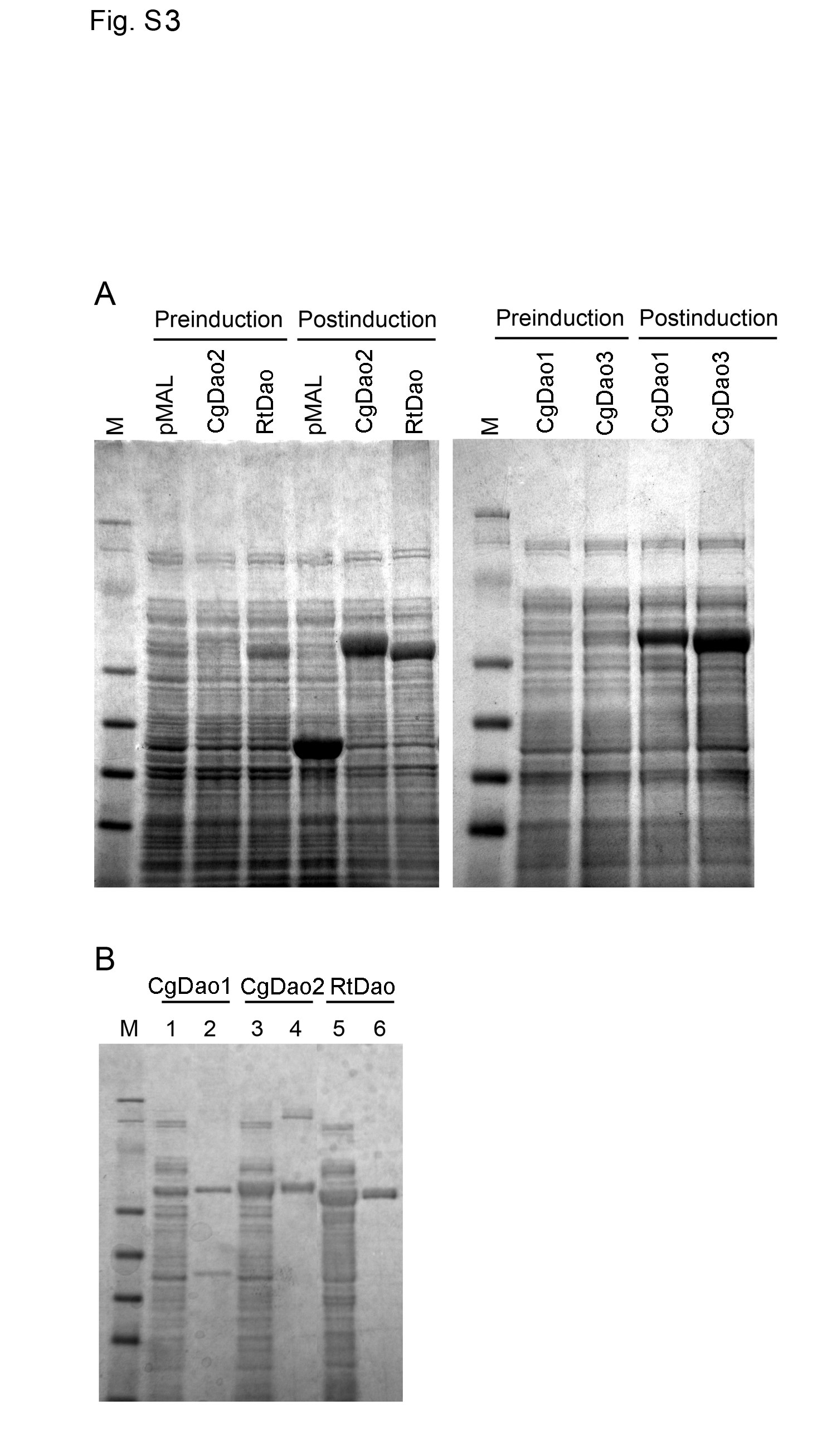

Supplement: S3 Fig — (A) E coli. crude protein extract was isolated, separated in a 4–12% NuPAGE gel and stained with Comassie Blue stain reagent. Twenty ug of Cgdao2 and RtDao and 30 ug of Cgdao1 and Cgdao3 post-induction samples were loaded in wells. Approximately the same amount of pre-induction sample was loaded in wells. (B) Purity of the Dao recombinant proteins after affinity chromatography. Protein was separated by a 4–12% NuPAGE gel and stained with Comassie Blue reagent. Samples 1, 3, and 5 contain crude extract (10ug) and samples 2, 4, and 6 contain purified sample (2ug). Cgdao3 was not shown due to lack of detectable enzymatic activity. (TIF) [file pone.0131865.s003.tif]

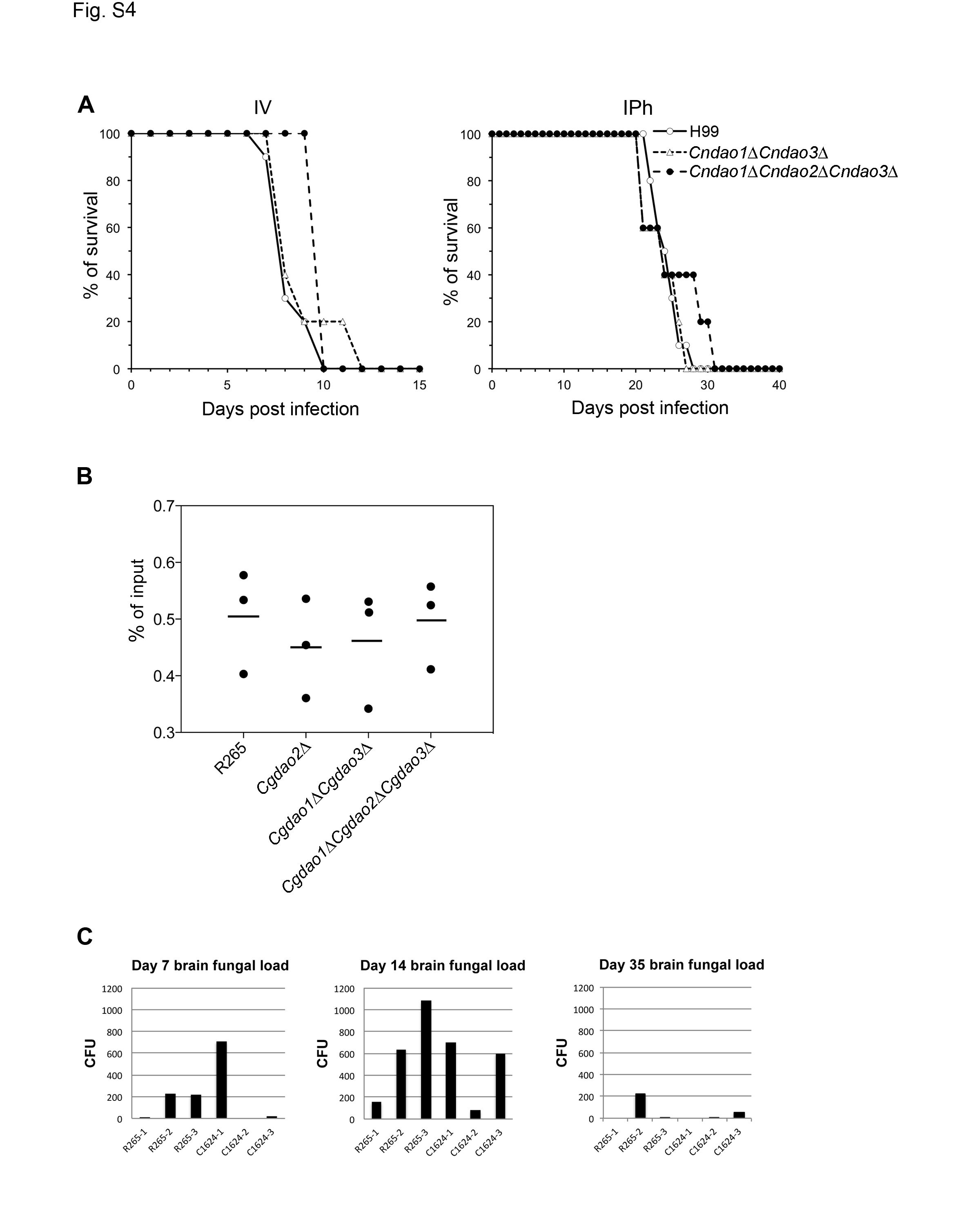

Supplement: S4 Fig — (A) Virulence H99 dao deletants is the same as the wild-type. BALB/c female mice (5 per group) were injected with various strains either by intravenous injection (IV) or intrapharyngeal aspiration (IPh) and the mortality was monitored. (B) Frequency of brain entrance is not reduced in the Cgdao triple deletant. Group of three mice each were infected intravenously with the indicated strains. Brains were isolated 3h post infection and the colony forming units in the brain were determined. Data is expressed as a percentage of the number of the CFU’s in the brain vs. the number of input cells. Bar: mean value of three mice. (C) Fugal burden in the brain of the mice infected by the Cgdao triple deletant is the same as the wild-type. Group of three mice each were infected by intrapharyngeal aspiration. Brains were isolated from the infected mice as indicated time and the colony forming units (CFU) per the brain were determined. (TIF) [file pone.0131865.s004.tif]
