# Supplementary material for: Differences between Cryptococcus neoformans and Cryptococcus gattii in the Molecular Mechanisms Governing Utilization of D-Amino Acids as the Sole Nitrogen Source
Source: PLoS One. 2015 Jul 1;10(7):e0131865. doi: 10.1371/journal.pone.0131865 (PMC4489021; doi:10.1371/journal.pone.0131865)
Supplement: S1 Table — (DOCX) [file pone.0131865.s005.docx]

**Table S1.** List of strains relevant to this study.

| Strains | Descriptions | Comment |
| --- | --- | --- |
| H99 | *C. neoformans* | Gift from Dr. John Perfect’s laboratory |
| C1577 | *Cndao1∆::NEO* | derived from H99 |
| C1579 | *Cndao2∆::NEO* | derived from H99 |
| C1581 | *Cndao3∆::NEO* | derived from H99 |
| C1634 | *Cndao1∆::NEO Cndao3∆::HYG* | derived from C1577 |
| C1643 | *Cndao1∆::NEO Cndao2∆::NAT Cndao3∆::HYG* | derived from C1634 |
| C1720 | *Cndao2∆::CgDAO2(p)::CgDAO2::HYG* | derived from C1579 |
| C1726 | *Cndao2∆::CgDAO2(p)::CnDAO2::HYG* | derived from C1579 |
| C1728 | *Cndao2∆::CnDAO2(p)::CgDAO2::HYG* | derived from C1579 |
|  |  |  |
| R265 | *C. gattii* | Gift from Dr. James W. Kronstad’s laboratory |
| C1584 | *Cgdao1∆::NEO* | derived from R265 |
| C1585 | *Cgdao2∆::NEO* | derived from R265 |
| C1588 | *Cgdao3∆::NEO* | derived from R265 |
| C1591 | *CgDAO2::HYG* | derived from C1585 by reconstituting  *Cgdao2∆::NEO* with *CgDAO2::HYG* |
| C1594 | *Cgdao1∆::HYG Cgdao2∆::NEO* | derived from C1585 |
| C1595 | *Cgdao2∆::NEO Cgdao3∆::HYG* | derived from C1585 |
| C1624 | *Cgdao1∆::HYG Cgdao2∆::NEO Cgdao3∆::NAT* | derived from C1594 |
| C1645 | *Cgdao1∆::NEO Cgdao3∆::NAT CgDAO2::BLE* | derived from C1624 by reconstituting  *Cgdao2∆::NEO* with *CgDAO2::BLE* |
| C1722 | *Cgdao2∆::CnDAO2(p)::CnDAO2::HYG* | derived from C1585 |
| C1724 | *Cgdao2∆::CnDAO2(p)::CgDAO2::HYG* | derived from C1585 |
| C1730 | *Cgdao2∆::CgDAO2(p)::CnDAO2::HYG* | derived from C1585 |
