# Supplementary material for: Differences between Cryptococcus neoformans and Cryptococcus gattii in the Molecular Mechanisms Governing Utilization of D-Amino Acids as the Sole Nitrogen Source
Source: PLoS One. 2015 Jul 1;10(7):e0131865. doi: 10.1371/journal.pone.0131865 (PMC4489021; doi:10.1371/journal.pone.0131865)
Supplement: S2 Table — (DOCX) [file pone.0131865.s006.docx]

| **Table S2. Primers relevant to the study** | |
| --- | --- |
| **To delete Dao1, Dao2, Dao3** |  |
| Hdao1A | CGTCGCTGACAAGTGGTCTA |
| Hdao1B | GCTAGTTTCTACATCTCTTCCGTGATTCGTTGTTTGTTGGTTCGTCT |
| Hdao1C | **CGCC**GCTCTCCAGCTCACATCCTCGACCTGGATGAGAACACAGTAGTC |
| Hdao1D | TAGAGCTGCAGGCGGTTACT |
| Hdao2A | CGTGACGATCCTTCTCCATT |
| Hdao2B | GCTAGTTTCTACATCTCTTCCGTGTAACGATATTTTGTGCGACGG |
| Hdao2C | **CGCC**GCTCTCCAGCTCACATCCTCACCGTGTTGTTTTCTCCTTTG |
| Hdao2D | AGTTTGGGTGGAGAGCATTG |
| Hdao3A | CGACGAAGAAAACCCCACTA |
| Hdao3B | GCTAGTTTCTACATCTCTTCCGTGCCATAATTGTTATAGTGTCATAAATTGCT |
| Hdao3C | **CGCC**GCTCTCCAGCTCACATCCTCTTGTTTGTTCATTTGTAAATGTCGA |
| Hdao3D | CGCCTTCCAAGAATCAGAAC |
| Rdao1A | TCGCAGGCTTTTTCTTTGTT |
| Rdao1B | GCTAGTTTCTACATCTCTTCCGTGATTATTTGTTTCGTCGTTGGTACG |
| Rdao1C | **CGCC**GCTCTCCAGCTCACATCCTCCCTGGATGGGAATACAGCAG |
| Rdao1D | AAGGGATATCCGACCAGTCC |
| Rdao2A | TATCAAACCGCCATTCAACA |
| Rdao2B | GCTAGTTTCTACATCTCTTCCGTGCACGATAGTTTGTGTGATAGTAGAAGAG |
| Rdao2C | **CGCC**GCTCTCCAGCTCACATCCTCACTGCGCTGTTTTCTTTTTTG |
| Rdao2D | GTGGCGAGCATTGAGTTGTA |
| Rdao3A | CAACCCCTCTTCATTCTCCA |
| Rdao3B | GCTAGTTTCTACATCTCTTCCGTGAATTTGATTGATGAACTCGCC |
| Rdao3C | **CGCC**GCTCTCCAGCTCACATCCTCGATGTTTATTCATAACGGATTACGTAAAT |
| Rdao3D | TACGAGGAGCGGAAGAAGAA |
| **To complement Dao deletants** |  |
| Rdao1A | TCGCAGGCTTTTTCTTTGTT |
| Rdao1B2 | GCTAGTTTCTACATCTCTTCCGTGACTCAGACGGGCACTAGGAA |
| Rdao2A | TATCAAACCGCCATTCAACA |
| Rdao2B2 | GCTAGTTTCTACATCTCTTCCGTGCGCAGAAGAAGGGAGTGTTC |
| Rdao3E | ACAGGACGCGATGCTAAACT |
| Rdao3B2 | GCTAGTTTCTACATCTCTTCCGTGCAAGGCAAAAACTGGAGAGC |
| Hdao1A | CGTCGCTGACAAGTGGTCTA |
| Hdao1H | CGTGCTGACGTTTTTCTTCA |
| Hdao2E | ACTGTTTCTTGGGACCGATG |
| Hdao2H | AGAGGTGGAGCCAACAGAGA |
| Hdao3A | CGACGAAGAAAACCCCACTA |
| Hdao3H | TGATCCCTCTCGGATTTTTG |
| **Gdp-Dao2 overexpression primers** |  |
| gpdh1A8 | TTGGTACCGAGCTCGGATCCCTGCACCAAACATGCCATAC |
| gpdh1B8 | TGTATTTATGCAAGTATATCCTAGAAGG |
| RDAO2-R265 | AGGATATACTTGCATAAATACAATGTCCTTTGATGCAGTAGTTATTG |
| B2.G418-Apa | CTATAGGGCGAATTGGGCCCGAGGATGTGAGCTGGAGAGC |
| gpdh1A9 | TTGGTACCGAGCTCGGATCCCCAATTTTGAAGCCAAAGGA |
| gpdh1B9 | TGTATTTATGCAAGTATACTCCTAGAAG |
| RDAO2-H99 | GGAGTATACTTGCATAAATACAATGTCCTTTGATGCAGTAGTTATTG |
| B2.G418-Apa | CTATAGGGCGAATTGGGCCCGAGGATGTGAGCTGGAGAGC |
| **Other GPD-Dao overexpression primers** |  |
| gpdh1A8 | TTGGTACCGAGCTCggatccCTGCACCAAACATGCCATAC |
| gpdh1B8 | TGTATTTATGCAAGTATATCCTAGAAGG |
| H1A | AGGATATACTTGCATAAATACAATGTCTCCTCCTTTAGACTCTTCTC |
| H1B | GCTAGTTTCTACATCTCTTCCGTGCGGCCGCGTGAGAGTGGCTGGATGTGA |
| H2A | AGGATATACTTGCATAAATACAATGTCCTTTGACGCCGTCGTTA |
| H2B | GCTAGTTTCTACATCTCTTCCGTGCGGCCGCAATCAACACCCTCGTCGTTC |
| H3A | AGGATATACTTGCATAAATACAATGGTAAAATACGACGCTGTCATC |
| H3B | GCTAGTTTCTACATCTCTTCCGTGCGGCCGCTGATCCCTCTCGGATTTTTG |
| R1A | AGGATATACTTGCATAAATACAATGTCTCCTTCTCTTGACTCTTCTC |
| R1B | GCTAGTTTCTACATCTCTTCCGTGCGGCCGCGGCTTGGGTGAGAGTAGCTG |
| R3A | AGGATATACTTGCATAAATACAATGGTGAAGTACGACGCTATCATC |
| R3B | GCTAGTTTCTACATCTCTTCCGTGCGGCCGCAGCAGGTTAATCGCTCGAAA |
| **Gene and promoter swap primers** |  |
| R2E1 | TTGGTACCGAGCTCGGATCCTATCAAACCGCCATTCAACA |
| R2F1 | AGTTTCTACATCTCTTCCGTGCGGCCGCCGCAGAAGAAGGGAGTGTTC |
| H2E1 | TTGGTACCGAGCTCGGATCCCGTGACGATCCTTCTCCATT |
| H2FX | TGTTGAATGGCGGTTTGATACGACGGTAGACGGGAGGTTT |
| R2Fx | AATGGAGAAGGATCGTCACGGAAGAGAGGATGGGTGAGTCG |
| H2E2 | CGTGACGATCCTTCTCCATT |
| H2F2 | AGTTTCTACATCTCTTCCGTGCGGCCGCAATCAACACCCTCGTCGTTC |
| H2F3 | CAATAACTACTGCATCAAAGGACATTAACGATATTTTGTGCGACGG |
| R2E3 | ATGTCCTTTGATGCAGTAGTTATTG |
| R2F2 | TAACGACGGCGTCAAAGGACATCACGATAGTTTGTGTGATAGTAGAAGAG |
| H2E4 | ATGTCCTTTGACGCCGTCGTTA |
| **qRTPCR primers** |  |
| Hdao2fr | CAATTACATTCGTCATCTCAGTTCAGA |
| Hdao2rv | AGAAAGACGGTAGCGGTGGAT |
| Hdao probe | CCGTGCGCGAGGCATCCC |
| Rdao2fr | AATTACATCCGTCATCTCAGTTCAGA |
| Rdao2rv | GAAGAGAGTCGGTAGCGATGGA |
| Rdao probe | CCGTGCGCGAGGCATCCC |
| **expression of Dao in pMAL-c5X** |  |
| RDao1A3 | AAC CTC GGG ATC GAG GGA AGG ATG TCT CCT TCT CTT GAC TCT TC |
| RDao1B3 | CCTGCAGGGAATTCGGATCC TCA AAT CCC AAT CGC CTT GC |
| RDao2A3 | AAC CTC GGG ATC GAG GGA AGG ATG TCC TTT GATGCAGTAGTTATTGG |
| RDao2B3 | CCTGCAGGGAATTCGGATCCCTATAATTTCGCTGACGTGCG |
| RDao3A3 | AAC CTC GGG ATC GAG GGA AGG ATG GTG AAG TAC GAC GCT ATC |
| RDao3B3 | CCTGCAGGGAATTCGGATCCCTAAAGCTTTGCCTTATTATCACTGC |
| RtDao2A1 | AAC CTC GGG ATC GAG GGA AGG ATG CAC TCT CAG AAG CGC GT |
| RtDao2B1 | CCTGCAGGGAATTCGGATCC CTACAACTTCGACTCCCGCG |
| **Primer for Northern probes** |  |
| Hdao1I | CGCCGTCTCGTTTACTTCTC |
| Hdao1J | GTCGCCTTGCAAAATAGAGC |
| Hdao2I | CGGCAAGAAGTTTGGTCACT |
| Hdao2J | TGCAGATCCAATACCATAGGC |
| Hdao3I | GAGGAAGTCCATGCACCATT |
| Hdao3J | ACCAACTCTCCGACCTCCTT |
| Rdao1I | CGCGGTCTCGTTTACTTCTC |
| Rdao1J | CTTGCAAAGCAGAGCAACAG |
| Rdao2I | CAAAAAGCTCAAATCGACTCC |
| Rdao2J | ACTTTCCTCCTCTCGCCTTC |
| Rdao3I | CCTACACGCTCAACACTCCA |
| Rdao3J | TTCTTCACGCACGCATCTAC |
